# Supplementary material for: Fidelity of hyperbolic space for Bayesian phylogenetic inference
Source: PLoS Comput Biol. 2023 Apr 26;19(4):e1011084. doi: 10.1371/journal.pcbi.1011084 (PMC10166537; doi:10.1371/journal.pcbi.1011084)
Supplement: S1 Text — Appendix A: MCMC details. Appendix B: Wrapping proposal vectors onto the hyperboloid. Appendix C: Impact of Normal prior on posterior. Appendix D: Closed form pf projection Jacobians. Appendix E: Algorithm Run Time. Appendix F: Datasets. Appendix G: Effective sample size. (PDF) [file pcbi.1011084.s001.pdf]

S1 Text

Appendix A MCMC details Our interest is forming posterior distributions of trees, which we achieve through MCMC sampling of the posterior distribution. MCMC is a classical method for sampling the posterior distribution $p(T|Y)$ given some data Y. Starting at an initial location in the posterior X, a new point X^* is proposed from a proposal distribution $g(X^*|X)$ and accepted or rejected according to the Metropolis-Hastings algorithm. A new proposal state X^* decodes a tree $T^* = \mathrm{NJ}(X^*)$ which is accepted with probability $\min(1, \alpha)$, where the acceptance ratio is

$$\alpha = \frac{p(Y|T^*)p(T^*)}{p(Y|T)p(T)} \frac{g(X|X^*)}{g(X^*|X)}$$

Importantly, it does not depend on the intractable component of the posterior $p(Y)$. Choosing a symmetric proposal $g(X^*|X)$, such as a Normal distribution, makes the last term simply cancel out. After a period of burn-in, points generated from this simple procedure compose autocorrelated samples from the targeted posterior distribution.

Method for initial tuning of the MCMC step size. A simple method for tuning the covariance matrix in MCMC to achieve a target acceptance rate a^* can be achieved as follows. Given an initial covariance matrix Σ, we scale it by a factor s that is tuned. Assume that increasing the step size decreases the acceptance rate and vice versa. We solve the following ordinary differential equation for the step size s:

$$\frac{ds}{da} = a - a^*$$

using the Euler method:

$$s_{n+1} = s_n + \eta(a_n - a^*). \tag{1}$$

The learning rate $\eta = (1 + n)^{-\lambda}$ decays to zero. This ensures that as $n \to \infty$ the step size is constant and the target distribution is sampled. We make the simple choice of the small multiple $\zeta = 0.1$ of the identity matrix as the initial covariance and choose $\lambda = 0.5$.

This method is similar to the adaptive scaling metropolis (ASM) algorithm [1] with a small difference. In Eq. 1, replacing the acceptance rate over all past states a_n by the acceptance probability of the current proposal α reproduces ASM. We find that using a_n leads to better performance for the warm-up phase, giving faster convergence and an acceptance rate closer to the target acceptance of 0.234.

Improved MCMC We make use of two modifications to the standard MH-MCMC algorithm. First, to improve mixing, Metropolis Coupled MCMC can use multiple chains that can swap intermittently through an MCMC move [2, 3]. The advantage of this is the ability to heat chains to move out of local optima more freely and it is routinely used in MrBayes. This is achieved by raising the acceptance ratio to a power given by a temperature parameter α^τ, $0 \leq \tau \leq 1$. We use four chains with temperatures $\tau_i = 1/(1 + \lambda i)$, where $i = 0, 1, 2, 3$ is the chain index and $\lambda = 0.1$ is fixed, as is done in MrBayes. This ensures that the first chain remains *cold* ($\tau_0 = 1$) and correctly samples from the posterior distribution.

The second idea is to adapt the covariance matrix of proposals using the Robust Adaptive Metropolis (RAM) algorithm [4]. The RAM algorithm is a variant of the Metropolis-Hastings algorithm that uses a covariance matrix to adapt the proposal distribution. During a warm-up phase, we tune the initially diagonal covariance matrix

simply by scaling it to achieve the target acceptance rate of 0.234, see SI 1. After the warm-up phase, we employ the RAM algorithm for the duration of the MCMC. Simultaneously, it tunes the acceptance rate to ensure computational efficiency.

MCMC proposals The specific proposal distribution we use for a sequence embedded at location $\boldsymbol{x}_i \in \mathbb{H}^d$, a Gaussian is sampled in the projection to $\mathbb{R}^d$: $\mathcal{N}(\phi^{-1}(\boldsymbol{x}_i), \Sigma_i)$. Since there are n taxa, the complete proposal lies in $\mathbb{R}^{nd}$. Here we use a Gaussian $\mathcal{N}(Y, \Sigma)$, where Y is the flattened vector of all taxa projections $\phi^{-1}(\boldsymbol{x}_i)$. The covariance matrix is initialised to a scalar multiple of the identity $\Sigma = \zeta I_{n\times d}$, with $\zeta = 0.1$, before being tuned by the MCMC algorithm. The sample points are then projected onto the hyperboloid exactly using Eq.1 (in main) and a tree is decoded.

Note that the priors are not in the sampling space. Instead, the Gamma-Dirichlet prior is on the tree, which induces a Jacobian adjustment, detailed in supporting information S4.

Appendix B Wrapping proposal vectors onto the hyperboloid. [5] present a method to wrap Euclidean vectors onto the hyperboloid. This allows Dodonaphy to sample vectors drawn from arbitrary distributions in hyperbolic space. We provide a brief outline of this method and use it for proposals within Dodonaphy to compare results.

The method has two steps, parallel transport of vectors tangent to the hyperboloid and then mapping exponentially from the tangent space onto the hyperboloid. Parallel transport takes a vector in one tangent plane $x \in \mathbb{T}_\nu\mathbb{H}^d$ and places it in another tangent plane $\mathbb{T}_\mu\mathbb{H}^d$ whilst conserving the vector's metric. It is given by:

$$\mathrm{PT}_{\nu\to\mu}(x) = x + \frac{\langle \mu - \alpha\nu, x\rangle}{\alpha + 1}(\nu + \mu)$$

where $\alpha = -\langle \nu, \mu\rangle$

Then, the exponential map wraps this vector in the tangent space onto the hyperboloid. It is constructed to preserve the vector's norm $||x||_{\mathcal{L}} = \sqrt{\langle x, x\rangle}$ as follows

$$\exp_\nu(x) = \cosh(||x||_{\mathcal{L}})\nu + \frac{x}{||x||_{\mathcal{L}}}\sinh(||x||_{\mathcal{L}}).$$

As an example, Dodonaphy samples Euclidean vectors from a Normal proposal distribution and wraps them onto the hyperboloid. This is instead of projecting the first coordinate of the hyperbolic vector. Like before, MCMC is run for 10^7 generations and all else is held equal with $\kappa = -1$, $d = 3$.

As expected from MCMC, the results of using these proposals are reasonably similar to projecting proposal vectors via with ϕ. Both the split frequencies (ASDSF of 0.004) and mean lengths are captured well, Figure 1. The similar results of this method demonstrate the possibility of using alternative proposals.

Appendix C Impact of Normal prior on posterior. Placing a prior on embedding locations, rather than tree samples avoids the Jacobian involved in MCMC for transforming from embeddings to trees. Here, we use a standard multivariate normal distribution $\mathcal{N}(\vec{0}, \Sigma)$ with covariance given by the d dimensional identity matrix. As before, Dodonaphy runs for 10^6 iterations including a 10^4 warm-up period using the tuning method presented above. It starts from the distances on the consensus tree from the golden run of MrBayes.

As expected, the tree length estimates vary from those given by the golden run, merely because they have different priors, Figure 2. More to the point, it demonstrates that there is no clear trend in trees becoming longer with higher dimensions. This suggests that when a Gamma-Dirichlet prior on trees is used, the trend in increasing tree length (in the main text) derives from the non-trivial Jacobian involved in the MCMC.

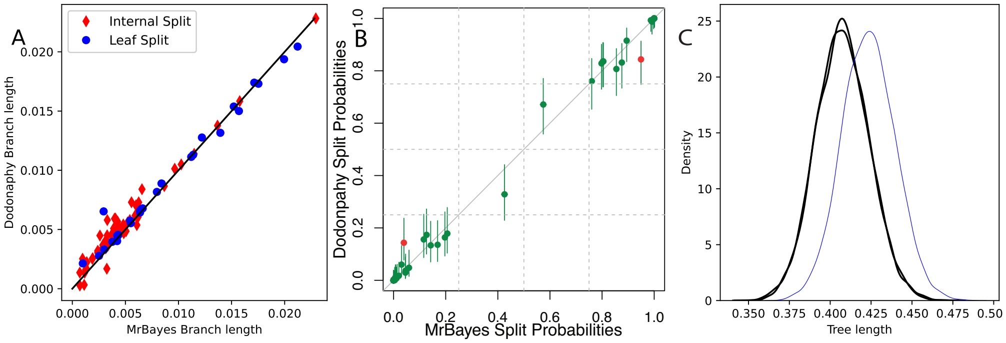

Fig 1. MCMC with wrapped Normal proposals compared to a MrBayes golden run. a) Mean split lengths. Leaf edges in blue circles, internal edges in red diamonds. b) Difference in split frequencies with Fréchet confidence intervals (red dots: MrBayes value not covered by CI). c) Tree length distribution (black: MrBayes and blue: Dodonaphy).

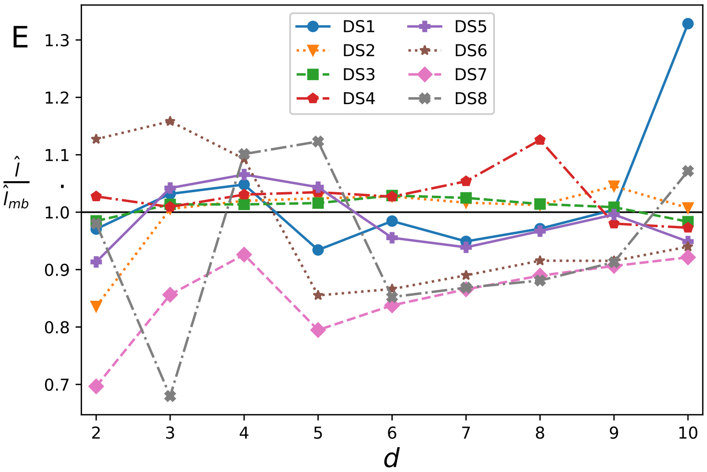

Fig 2. Median tree length estimates compared to MrBayes over varying dimensions with a Normal prior on embedding locations.

Appendix D Closed form of projection Jacobians The Jacobian of the first projection $\phi : \mathbb{R}^d \to \mathbb{R}^{d+1}$ is non-square because it attaches an extra dimension to each taxon. For a taxon with stored location $\boldsymbol{x} \in \mathbb{R}^d$, it takes the form

$$\frac{\partial \phi(\boldsymbol{x})}{\partial \boldsymbol{x}} = \begin{bmatrix} \boldsymbol{x}/\hat{\boldsymbol{x}} \\ I_d \end{bmatrix} \in \mathbb{R}^{d+1} \times \mathbb{R}^d$$

For the second transformation from the hyperboloid to the pairwise distances, the Jacobian of the distance between two points $\boldsymbol{x}, \boldsymbol{y} \in \mathbb{H}^d$ is adapted from [6]:

$$\frac{\partial d(\boldsymbol{x}, \boldsymbol{y}, \kappa)}{\partial \boldsymbol{x}} = \frac{1}{\sqrt{-\kappa((\boldsymbol{x} * \boldsymbol{y})^2 - 1)}} \Big(\frac{\sqrt{||\boldsymbol{y}||_2^2 + 1}}{\sqrt{||\boldsymbol{x}||_2^2 + 1}} \boldsymbol{x} - \boldsymbol{y} \Big).$$

It is clear that the distance between $\boldsymbol{x}$ and $\boldsymbol{y}$ does not depend on a third point $\boldsymbol{z}$:

$$\frac{\partial d(\boldsymbol{x}, \boldsymbol{y}, \kappa)}{\partial \boldsymbol{z}} = 0.$$

Appendix E Algorithm Run Time The three most time-consuming algorithms of Dodonaphy's MCMC are the likelihood calculation $O(nL)$, neighbour-joining $O(n^3)$ and the pairwise distance computation $O(n^2 d)$ [6]. We run Dodonaphy on Dell PowerEdge R640 or R740 servers with dual Intel(R) Xeon(R) Gold 6240 CPUs running at 2.60GHz. For each run, we record the duration per chain at each generation t over a range of taxa sets n with varying unique site patterns L and various embedding dimensions, Figure 3. Adding the three main algorithms together, the best fit with standard deviations is $an^3 + bLn + cn^2 d$ with $a = (5.086 \pm 0.766) \times 10^{-5}$ ms, $b = (2.529 \pm 0.205) \times 10^{-4}$ ms and $c = (6.884 \pm 0.661) \times 10^{-4}$ ms. Assuming three dimensions and taxa with $L = 1000$ unique sites, this means that the majority of the time is spent on neighbour joining when there are more than 237 taxa.

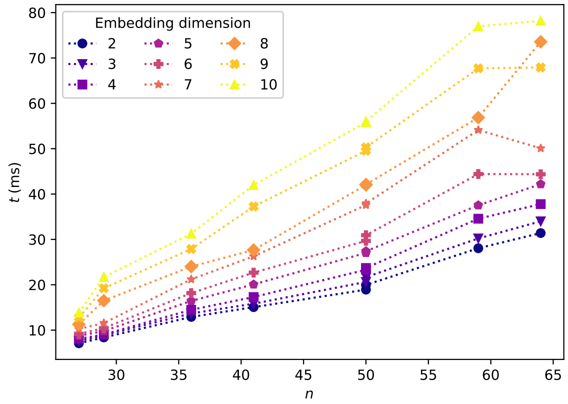

Fig 3. Mean time per generation. For one chain of Dodonaphy over each embedding dimension. Note that two datasets with different alignment lengths contain $n = 50$ taxa.

Overall, the expected performance complexity is between quadratic and cubic in the (finite) number of taxa. Improvements to the NJ algorithm would yield the most significant speed-up as the number of taxa grows since, at $O(n^3)$, it is asymptotically the bottleneck algorithm. For example, there are fast NJ implementations suitable for tens or hundreds of thousands of sequences [7, 8]. Similarly, BEAGLE provides a fast implementation of the likelihood computation [9, 10] that could improve CPU time.

Table 1. Dataset details.

Label	n	sites	Type of data	Treebase
DS1	27	1949	rRNA, 18s	M2017
DS2	29	2520	rDNA, 18s	M2131
DS3	36	1812	mtDNA, COII (1–678), cytb (679–1812)	M127
DS4	41	1137	rDNA, 18s	M487
DS5	50	378	Nuclear protein coding, wingless	M2907
DS6	50	1133	rDNA, 18s	M220
DS7	59	1824	mtDNA, COII and cytb	M2449
DS8	64	1008	rDNA; 28s	M2261

Appendix F Datasets Table 1 details the datasets used in this study.

Appendix G Effective sample size As an alternative to the ASDSF, we also computed the difference in split frequencies between a golden run of MrBayes to each simulation and compared it to the expected difference at a given effective sample size (ESS) [11]. We fixed the expected sample size at 625 to compute the expected difference between runs. We plotted the proportion of splits performing better than this expected difference in Figure 4. These results correlate with the main ASDSF results, Fig 3 and Fig 4 in the main text. As in Fig 4a, the impact of embedding dimension was approximately constant except for two dimensions which produced poorer splits. For the curvature (Fig 3a), using a flatter space (a more negative log minus curvature) similarly produced poorer split effective sample sizes. The reduced ASDSF performance in more curved spaces (e.g. $\kappa \to -10^4$) was not as evident when looking at the split ESS, Fig 4b. The best performance was for a curvature approximately in the range of -100 to -1, which is consistent with Figure 3a.

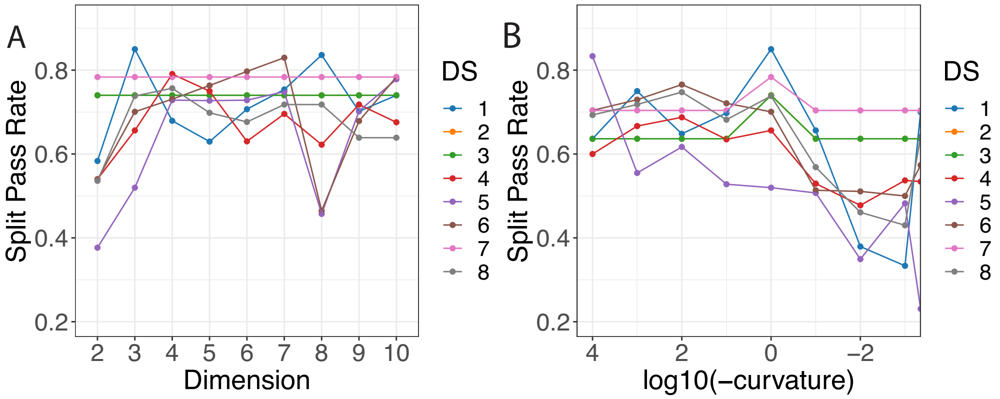

Fig 4. Proportion of splits passing an effective sample size of 625 across a) embedding dimension (fixed curvature of -1) and b) curvature (fixed dimension 3).

References

1. Atchadé YF, Rosenthal JS. On adaptive Markov chain Monte Carlo algorithms. Bernoulli. 2005;11(5):815–828. doi:10.3150/bj/1130077595.

2. Geyer CJ. Markov Chain Monte Carlo Maximum Likelihood. Interface Foundation of North America; 1991.

3. Altekar G, Dwarkadas S, Huelsenbeck JP, Ronquist F. Parallel Metropolis Coupled Markov Chain Monte Carlo for Bayesian Phylogenetic Inference. Bioinformatics. 2004;20(3):407–415. doi:10.1093/bioinformatics/btg427.

4. Vihola M. Robust Adaptive Metropolis Algorithm with Coerced Acceptance Rate. Statistics and Computing. 2012;22(5):997–1008. doi:10.1007/s11222-011-9269-5.

5. Nagano Y, Yamaguchi S, Fujita Y, Koyama M. A Wrapped Normal Distribution on Hyperbolic Space for Gradient-Based Learning. In: International Conference on Machine Learning. PMLR; 2019. p. 4693–4702.

6. Chowdhary K, Kolda TG. An Improved Hyperbolic Embedding Algorithm. Journal of Complex Networks. 2018;6(3):321–341. doi:10.1093/comnet/cnx034.

7. Wheeler TJ. Large-Scale Neighbor-Joining with NINJA. In: Salzberg SL, Warnow T, editors. Algorithms in Bioinformatics. Berlin, Heidelberg: Springer; 2009. p. 375–389.

8. Wang W, Barbetti J, Wong T, Thornlow B, Corbett-Detig R, Turakhia Y, et al. DecentTree: Scalable Neighbour-Joining for the Genomic Era. bioRxiv. 2022;.

9. Ayres DL, Darling A, Zwickl DJ, Beerli P, Holder MT, Lewis PO, et al. BEAGLE: An Application Programming Interface and High-Performance Computing Library for Statistical Phylogenetics. Systematic Biology. 2012;61(1):170–173. doi:10.1093/sysbio/syr100.

10. Ayres DL, Cummings MP, Baele G, Darling AE, Lewis PO, Swofford DL, et al. BEAGLE 3: Improved Performance, Scaling, and Usability for a High-Performance Computing Library for Statistical Phylogenetics. Systematic Biology. 2019;68(6):1052–1061. doi:10.1093/sysbio/syz020.

11. Fabreti LG, Höhna S. Convergence assessment for Bayesian phylogenetic analysis using MCMC simulation. Methods in Ecology and Evolution. 2022;13(1):77–90.
