# Supplementary material for: Fidelity of hyperbolic space for Bayesian phylogenetic inference
Source: PLoS Comput Biol. 2023 Apr 26;19(4):e1011084. doi: 10.1371/journal.pcbi.1011084 (PMC10166537; doi:10.1371/journal.pcbi.1011084)
Supplement: Attachment — Submitted filename: Response to reviewers.pdf [file pcbi.1011084.s002.pdf]

Reviewer #1:

> Summary: This paper examines the use of hyperbolic embeddings for Bayesian phylogenetic inference and develops a software package which implements this technique.
>
> Strengths: A number of common test datasets are used to study what sorts of hyperbolic spaces are best for inference, and attention is paid to how MCMC moves through the space and how the existence of the tree leaves residual discontinuities. All of this is useful for future theoretical and empirical studies.
>
> Weaknesses: I find the presentation of material in the paper a bit disjointed and the promises of gradients (differentiable embeddings) oversold.
>
> My overall impression is that the authors have implemented an interesting piece of software and that the paper is a useful examination of the suitability of hyperbolic spaces for Bayesian inference of phylogenies. I have attempted to suggest some reorganization that I believe would help smooth the manuscript out.

Major issues

> I do not have an major issues with the paper.

Minor issues

> - Some additional clarity regarding the structure of the transformations of the inferred space would be useful.

We added a section called "Dodonaphy Algorithm" which clarifies the transformations involved in the algorithm. Algorithm 1 itself is more detailed in how the data a transformed between spaces. Figure 1 also better illustrates the projection transform phi.

> - I find myself somewhat unclear what the point of the first paragraph in the section "Embedding Fidelity" is, and what is being said about the order of operations.

See the next comment.

> - It is said that the authors "test Dodonaphy’s capacity for MCMC starting directly from sequence data." I am not sure what is meant. If the point is that no middle-man program is needed, and that Dodonaphy can handle the neighbor joining, likelihood, and transformations, I think that could be stated more clearly.

This first paragraph has been tweaked to make the setup for this section clear. Yes, no middle-man program is needed. In this section, Dodonaphy is not fed an initial tree, instead, it generates an embedding from the sequence data.

> - Is the order of operations to construct the initial embedding sequences->D->NJ tree->tree distance matrix->embedding or sequences->D->embedding? If the latter, does starting with a potentially non-additive embedding increase burnin?

In the section "Embedding Fidelity" the order is the latter: sequences->D->embedding. Yes, this leads to a longer burn-in, but the point is to demonstrate the ability of Dodonaphy to start from this sub-optimal state. As mentioned at the end of this section, the following sections initialise as sequences->MrBayes->consensus tree->tree distance matrix->embedding. This is in a heuristic to reduce burn-in, although as you point out, one could also start from the NJ tree, which would be considerably faster. Dodonaphy exposes an API (Option --start) to read in a tree in nexus format for the initial distances as desired, which can be set to NJ.

> - I don't follow the point about local changes with curvature, though it seems important. Does not changing one node's location change all distances involving that node and thus change potentially many branch lengths, regardless of the space?

This refers to local "changes in the (embedded) distances between taxa", rather than local changes in the topology. Yes, it has a small (local) impact on all the distances. This is opposed to thinking of local as "changes in a small subset of the tree topology". The effect of more (negative) curvature is to spread points further from the origin (see the section hyperboloid model), making the points further from each other, or more local. We have added a clarification in the "Posterior Landscape" section.

> - I find the section "MCMC Proposals" rather hard to follow. It is a mix of information about the specific proposal distribution (and its tuning) and the space of the operations. And the variable X is overloaded.

We have simplified this explanation (now in the supporting information S1).

> - I would suggest focusing on the space in which moves are made (rather than specifics of the move), that is, noting that moves take place in R^nxd, for tips whose locations live in H^d, while the priors and likelihood exist in the space of trees with branch lengths.

We have made a section titled "Dodonaphy Algorithm" that focuses on the algorithm and the spaces involved and noted the difference between the sampling space and the space that the prior works in. The spaces of the moves are now more explicit in both the algorithm and text. Afterwards, we give an overview of the specifics of each move and place the details in supporting information S1.

> - I believe it would also be helpful to either briefly discuss the Jacobian here or forward-reference the fact that the Jacobian will be discussed in more detail later. The Jacobian, and the related difficulties with placing priors on branch lengths when using hyperbolic embeddings, seem to me to be an important finding of the paper that is otherwise relegated to the discussion and the supplement.

We have introduced and forward-reference the Jacobian in the "experiments" section of the "Materials and methods".

> - It is said that, "This improvement saturates at about kappa = –1, indicating that the error to the four-point condition delta is negligible compared to the branch lengths." This seems important but I am not sure what "negligible compared to the branch lengths" means.

The error on the four-point condition \delta is a length, which when compared to the set of branch lengths on the tree is small, making practically no difference to the result. In the case of a consistent tree, we could say that \delta << d(x, y) for all taxa locations x, y. We have added "practically" as a qualifier to this claim.

Choice and dimensionality of the hyperbolic space

> - The sections "Embedding Curvature" and "Embedding Dimension" are very useful and sort of undersold beforehand.

We have mentioned this part of the work in the abstract.

> - These sections use 8 empirical datasets and a wide range of both kappa and d (leading to something near 20 analyses per dataset I believe) to come up with good choices of the hyperbolic space. This is a very useful contribution that this paper makes to any future investigations of hyperbolic embeddings in Bayesian phylogenetics.

Thank you, we also believe this analysis is useful for future work. We have added a clause in the conclusion to reflect this remark.

> - However, until the above 2 sections are read, the choices of dimension and curvature made seem rather arbitrary. I think it would be useful to state clearly in "Embedding Fidelity" that the choices of kappa and d have been informed by extensive analysis.

We have added such a statement in the first paragraph of the "Embedding Fidelity" section. We have also made this point clearer in the introduction to the "Results" by adding a sentence on this and moving details of the ASDSF into the section "Embedding Fidelity".

> - It might also bear repeating in "The Hyperboloid Model" that the choice of both kappa and d will be investigated empirically on a number of real datasets.

We have added a sentence to say this.

> - I think that the section "Tree Length Continuity" would be much more useful as a section in the methods, specifically as a subsection of "Tree Decoding." It provides links between kappa and delta that I found myself wanting when reading "Tree Decoding" and it currently interrupts the flow of the otherwise empirically-focused results.

We have moved the section "Tree Length Continuity" as asked and removed the, now out-of-place, final sentence. We have also switched the two "Result" subsections "Embedding Dimension" and "Posterior Landscape" for reading flow.

Superfluous text:

> there are several sections where the paper could be streamlined by moving material to the supplement. I do not wish to force the authors to write the paper as I would have written it, so the authors should feel free to ignore these suggestions if they do not believe them to be helpful.
>
> - Most of the section "Differentiable Embeddings" strikes me as extraneous because the conclusion of the first paragraph is that we are still stuck with a tree that gets in the way of derivatives. Without sketching some possible way around this, I must confess I don't see much point to the section.

See point [A] below.

> - I don't think lines 381-383 ("If the distances... substitutions per site.") are particularly necessary. If we can't guarantee ultrametric distances, then I do not see the point of clock models. And if we can, then clock models and clock rates are standard enough that a statement like "ultrametric distances would permit the use of molecular clock models" would suffice, no?

These lines have been shortened as suggested.

> - While I applaud the authors for being very thorough in explaining their MCMC setup, I am not sure that all of the provided information is required in the main text.

The details of the MCMC have been moved to the supplemental information.

> - Beyond stating the prior, posterior, and likelihood, I think lines 99-103 can largely be removed. The Metropolis-Hastings algorithm is rather standard.

We have moved these sections to the supporting information. Whilst the MH algorithm is standard, enmeshing it with hyperbolic encodings and decodings is not standard, so we have kept these details in the SI.

> - The section "Improved MCMC" could be moved to the supplement and replaced with a sentence like, "We use Metropolis-Coupled MCMC with 4 chains and an adaptive multivariate normal proposal." Adaptive proposals are common in all areas of MCMC and MC^3 should be familiar to most of the target audience.

We have moved this section to the supplement information and replaced it with the suggestion in the section "Bayesian Phylogenetics".

> - Related to the point I raised above, I find the promise in the abstract of discussing differentiable embeddings to be somewhat misleading. The discussion amounts, unless I am missing something, acknowledging that it is currently impossible. While I agree that it would be nice if we could have/use differentiable embeddings, unless the authors can sketch some solution around the problem (the tree) I would prefer the authors either remove this statement from the abstract or qualify it more transparently. "Prospects" does not convey that this is a very theoretical discussion indeed.

[A] We are currently working on a promising solution for differentiable embeddings. The idea is to use soft relaxations of the sorting operator to implement a differentiable version of neighbour joining. However, these details remain outside the scope of this paper. Accordingly, we have tightened this discussion point to reduce its superfluousness whilst also mentioning how this prospect will be actualised.

> - In the section "Adding Taxa To an Existing Phylogeny," I am unclear as to whether this is being suggested as an algorithm to place a new sequence into an entire posterior distribution of trees (very cool!) or for point estimates (which is still useful, if perhaps at the far edge of the scope of the paper).

We have clarified that this is to embed the new sequence into an entire posterior distribution.

Figures:

> there are a number of small improvements that could be made to the figures that would, in aggregate, make the paper easier to read.
>
> - The fonts for the figure axes are all very small and hard to read, please increase the font size by a large amount.

The smallest font sizes for the figures in the main text have been increased. Additionally, we have simplified some of the figure axes for readability. The x-axis of fig 3 uses log labelling and some unnecessary tick labels have been omitted (fig 4, 5).

> - Fig 2: I think a legend in the figure explaining diamonds versus circles would help. Regardless, though, what is the difference between transparent and solid diamonds? Are the solid ones multiple runs with the exact same value?

In figures 2a and 7a, we have added a figure legend explaining that the circles are for branches to leaves and diamonds are for internal edges.

> - Fig 2B is pretty but not as easily interpretable as a standard split-split plot, which I would advocate for (but am not requiring as a prerequisite for acceptance)

We have changed figures 2b and 7b to standard split-split plots and included confidence intervals.

> - Fig 4: the green diamond is very hard to tell apart from the black dots, at least on my screen

The green diamonds have been enlarged.

> - Fig 7: the caption should include "L"

Fig 7 "Mean time per generation" (now Fig 9) does not include the alignment length L since it is not used in the figure. The predominant calculation is neighbour joining O(n^3) not the likelihood calculation O(nL), so the most visually informative x-label is simply the number of taxa n.

> - Fig 9 has no legend

We have added a legend.

> - For figures comparing "true" to estimated branch lengths, I think that putting truth on the x-axis would more clearly communicate that branch-lengths are being over-estimated

These axes have been swapped, Fig 2c and 7a.

Typos and other small comments

> - Author list: Fourment is not capitalized

Fixed

> - l 24 typo: missing period in "move Recent"

Fixed

> - l 31 this may be overly pedantic, but there are plenty of Bayesian machine learning techniques, perhaps something like "as opposed to previous use of machine learning techniques in

> Bayesian phylogenetics" would be more appropriate?

Fixed

> - l ~51 I would advocate for replacing "the posterior probability is the probability of a tree and evolutionary model parameters" with something like "the posterior distribution is the joint posterior distribution of the tree and evolutionary model parameters"

Changed

> - l ~51 I find the use of psi for data very jarring, especially because (upper case) Psi is often used for the tree parameter. I hate to dictate notation but I would really appreciate the use of something more standard like y (lower or upper case, bold or not) or D.

Changed to capital Y since D and y are already used.

> - l 64 "it only computes" -> "it only requires computing"

Fixed

> - l 92 I can see how this guarantee is helpful once we have a tree, but wouldn't we risk running into trouble if we naively used the distance matrix instead of something based on a valid tree?

Yes, indeed a random distance matrix is not guaranteed to satisfy the four-point condition. However, hyperbolic space offers arbitrarily close approximations to this guarantee. This motive is now added to introduce this section.

> - l ~99 "computing the posterior" -> "sampling from/approximating the posterior"

Fixed

> - l 103 "compose samples from the targeted posterior distribution" -> "compose autocorrelated samples from the targeted posterior distribution"

Fixed

> - l 105 "original MCMC algorithm" -> "the original/basic/standard MH-MCMC algorithm"

Fixed

> - l 132 broken reference

Fixed

> - l 145 compression is such a standard approach that I don't think you have to say it is being done

Removed

> - l 156 I wonder if the authors might comment on their choice of the ASDSF < 0.05 threshold when they (e.g. Fourment et al. (2020)) have previously advocated for more stringent ones (in that case, RMSD < 0.01, comparable to, if more stringent than, ASDSF < 0.01)?

All these thresholds are arbitrary cut-offs as some measure of similarity between tree distributions. Both ASDSF < 0.05 and 0.01 are typically used and the actual values are visible in figures 3 and 5.

> - l 163 Given that convergence in likelihoods does not directly address convergence in treespace (e.g. Harris et al. (2021)), and that Figure 2B does imply convergence in trees, why cite Figure 2A and not 2B here?

This reference is updated to Figure 2b.

> - l 272 Some readers might be more familiar with this under the name RF distance

The manuscript now also refers to this as the Robinson-Foulds distance when first introduced in the "Posterior Landscape" section.

> - l 291 I believe that 5.086±0.766×1e−05 ms means (5.086±0.766)×1e−05 ms? This is not immediately clear to me but it lines up with the numbers in Fig 7

Yes. Fixed

> - l 313 I don't think I see why that in particular would be the bottleneck, would it not simply make O(dn^2) into O(n^3) like Neighbor Joining?

Yes, the computational time would become O(n^3) equivalent to neighbour joining. However, the memory requirements of the embedding will scale as O(ndL), which is always linear in the number of taxa in hyperbolic space. In Euclidean space, the memory becomes quadratic in the number of taxa O(n^2L)! A comment to refine this point is added to this section.

> - l 336 this sounds more like an MCMC convergence issue to me, no? shouldn't the MH step correct for that?

Yes, this is a convergence issue and you're right that the MH correction should account for any match between the proposal and target distribution. The manuscript now better reflects on how this convergence issue is attributed to the error in the four-point condition in close-to-Euclidean spaces.

> - l 344 It seems to me that the finding that changing the proposal also ameliorates this issue bears mention here too

We use a different proposal in supporting information S2 but it does not ameliorate the issue. The total length estimates are also over-estimated, fig 7c. This is consistent with the fact that both proposals undergo a Jacobian transformation that is not included. Using a different prior ameliorate the overestimation, which is what this section ("fidelity of embedded MCMC") builds towards.

> - l 344 It seems like it should be possible to place sane priors on the pairwise distances which should also obviate the problem and might be more interpretable than Normals in the embedding space? For example, two tips can't be separated by more than n-1 edges which would induce a Gamma(branch_length_rate,scale=n-1) distribution, and must be separated by at least 2 edges, which would yield a Gamma(branch_length_rate,scale=2) distribution, so something sane would lie between those and could still be parameterized implicitly in terms of a tree length (as a Gamma tree length and Dirichlet(1) edge proportions recovers exponential distributions on the branch lengths).

Yes, it may be more sensible to put priors on distances rather than embedding locations, however, we chose this Normal prior in the appendix to pinpoint the Jacobian as the source of the bias in higher dimensions. When sampling in the same space as the prior no Jacobian adjustment is required and we see that the long tree issue resolves itself. However, in the main text, we chose to implement the same Gamma-Dirichlet prior as in MrBayes for a cleaner comparison.

Putting a prior on the branch lengths is a good idea since we know the Jacobian between the embedding space and the pairwise distances. However, as pointed out, there's still the unresolved issue of the scale of the Gamma distribution. We agree it should lie between 2 and n-1, but it's difficult to go any further than this because the expected number of branches on the path between two tips will depend on the tree topology.

The last comment here, in brackets, on using a Gamma-Dirichlet prior is the prior that we do implement. This prior is on the pairwise distances which is not the same space that we sample points in (the tangent space to the hyperboloid), hence we need the Jacobians in supporting information S4. Regardless, we still need the non-trivial Jacobian for the change of variables going into tree space (by neighbour joining). We change variables from the target (tree) space back into either the pairwise distances or the locations on the hyperboloid (well, tangent space thereof) and the sample a new state. Current Bayesian phylogenetic software sample tree topologies separately from the continuous parameters and thus don't have to worry about this Jacobian.

> - l 351 extra space before "."

Fixed

> - l 394 I don't see why this is a problem? Most phylogenetic algorithms don't distinguish left from right, including, as I understand it, NJ. But identical sequence-induced symmetries do seem relevant, and I believe at least some of these DS do include identical sequences.

Isometries of the embedding don't lead to problems in the MCMC, as you questioned. This section is rewritten to emphasise that isometries present an opportunity, not a hindrance.

> - l 412 It also opens up potentially easier ways to diagnose MCMC convergence, give or take the potential issues induced by the symmetries.

By looking at a Normal prior in the embedding space, the MCMC does show convergence to a tree length that does not increase with dimension (supporting section SI5).

> - Fourment, Mathieu, et al. "19 dubious ways to compute the marginal likelihood of a phylogenetic tree topology." Systematic biology 69.2 (2020): 209-220.

This reference has been added in the introduction.

> - Harrington, Sean M., Van Wishingrad, and Robert C. Thomson. "Properties of Markov chain Monte Carlo performance across many empirical alignments." Molecular biology and evolution 38.4 (2021): 1627-1640.

This article was referenced on line 9.

Reviewer #2:

> In this manuscript, the authors proposed a novel phylogenetic tree inference method, Dodonaphy. The first key idea of Dodonaphy is to explore phylogenetic trees in an embedded space. This approach makes it possible to perform neighborhood search, which has been done by discrete operations such as NNI, by continuous operations. The second idea is the utilization of hyperbolic space. This space is more suitable for representing phylogenetic trees than the Euclidean space. The author confirmed that Dodonaphy reconstructs phylogenetic trees that is nearly equivalent to those of MrBayes in eight different data sets.
>
> I found that the idea is exciting, and the assertion is well confirmed in various experiments. However, the authors have neither made any new biological discoveries with this software nor presented the possibility of such discoveries. This is not consistent with the scope of the PLOS Computational Biology journal. At the very least, the authors should discuss what advantages Dodonaphy has over MrBayes, or could have in the future.

The ability to "Navigating Tree Space" in a completely new way presents opportunities for embedding methods over the state-of-art method, e.g. MrBayes. State-of-art methods navigate tree space using SPR and NNI moves, however, the revised section on "Navigating Tree Space" illustrates how multiple SPR moves (and hence NNI) moves can be done at once. This could present advantages in dealing with large phylogenies where it may take too many NNI (or even SPR) moves to significantly change tree topology and avoid local optima.

Another advantage that we finish on in the discussion is the future possibility of differentiable optimisation over tree space. This technique is something we are working on to open up a whole host of gradient-based methods to work on the discrete space of trees. The work in this paper lays a foundation for this branch of phylogenetics, which we believe holds promise for Maximum Likelihood, Maximum A Posteriori, Hamiltonian Monte Carlo and Variational inference directly in a representation of the space of trees.

Reviewer #3:

> Dear authors,
>
> I reviewed your paper with the title "Fidelity of hyperbolic space for Bayesian phylogenetic inference". Overall, I found the paper a bit hard to digest. My background is in computational phylogenetics, so the hyperboloid model approach was very dense and challenging to digest. Similarly, I can see readers less familiar with phylogenetics to stumble completely over the very briefly mentioned but not introduced concepts (e.g., NJ, JC, NNI and SPR). For a revised version, it would definitely be very helpful if the authors could explain all necessary concept more easily.

We have revised many parts of the manuscript to increase readability. We have revised the introduction to the hyperboloid model with the aim of making it more approachable. The figures have been reordered so that the first figure is an illustration of the model, which should help readers understand the heart of the model earlier on. We added further introductions to the concepts of NNI, SPR, JC69, and NJ.

> The ADSF is not a good measurement to compare between runs. The problem is when there are many splits that are considered, some strong outliers splits are overlooked because the average of the differences is taken. However, a split could be completely present in one analysis and completely absent in another. More recent work uses either the maximum deviation from the expectation (https://doi.org/10.1111/2041-210X.13727) or the Monte Carlo error of trees (https://doi.org/10.48550/arXiv.2109.07629). It would be better to use one of these approaches instead.

We acknowledge this weakness of the ASDSF and so computed the deviation in split frequencies and determined what proportion of splits deviate less than the expected split frequency deviation for a given ESS (625) as in the first link (Fabreti 2022). We added a section in the supporting information (S7) showing these results. They generally correlate with the ASDSF results in figures 3 and 4. Readers are directed to these additional results in the "parameter analysis" section, which notes the similarities and differences in its findings.

In the section embedding fidelity, we also report the Monte Carlo error of trees for the simulation presented in figure 2, as in Magee 2022.

> What is the reason that Dodonaphy overestimates the tree length? Aren't the models identical? If not, it would be important to show an example with identical models so that you can validate your implementation against MrBayes. If the models are identical, then you must find the reason for this discrepancy because it is likely either a bug in the likelihood computation or the MCMC algorithm.

The likelihood and prior models are identical. The only difference is the Jacobian between the sampling (hyperbolic) space and the target (tree) space. This point is outlined in supporting information S3 where we put the prior on the embedding space so that the Jacobian is unwarranted. In this case, overestimation disappears. We could use this as our main figure, but prefer to keep exactly the same prior as MrBayes in the main text.

We have verified that the likelihood and prior computation produces the same result as MrBayes on a test tree from MrBayes' output.

> I wonder how much of these results depend on the chosen datasets. In my experience, these datasets are in fact not that challenging. MrBayes performs extremely well on these dataset, so improvements are perhaps not that necessary. This could also explain your observation of only needing few dimensions for the embedding. However, there are many challenging datasets where MrBayes fails, see for example https://doi.org/10.1093/molbev/msaa295 Perhaps you can identify a few (~5) datasets were MrBayes has problems for convergence and apply your approach instead.

These datasets have previously been used numerous times as a benchmark for new methods, which is why we choose these datasets. Using a standardised set of datasets for testing new methods provides a more clear basis for comparison. The fact that MrBayes performs well on these datasets, especially after 10^9 generations gives us a good approximation of the posterior that we can compare to. Whidden and Matsen's 2015 paper Quantifying MCMC Exploration of Phylogenetic Tree Space illustrates some of the challenges in these datasets. Their second and third figures highlight how some of these datasets contain different clusters of tree topologies with very few SPR moves that can move between these clusters.

> Why would higher dimensions affect the tree length? Shouldn't the MCMC acceptance ratio alone influence the tree length? This sounds to me suspiciously as if there is a bug, perhaps in the likelihood if it doesn't correctly use the branch lengths.

We have tested explicitly that the same prior and likelihood are computed by MrBayes on a given tree on test data. In the discussion on lines 344-359 we attribute this discrepancy to the Jacobian involved between the sampling space (hyperbolic space) and the target space (tree space). The Jacobian of the final transformation by NJ is difficult to include since it is a non-differentiable operation.

> I disagree with your discussion paragraph starting at line 323. Even if a run with a lower dimension is stuck in a local optimum, this should not happen for repeated runs (at least not the same optimum). You should convince me and other readers that the inferred tree length posterior distributions are indeed correct. Only then makes it sense to speculate in the discussion about the reason why it is overestimated or underestimated. If the embeddings are only used as MCMC proposals, then all MCMC runs should converge to exactly the same posterior distribution (if run long enough)? Can you show if they do or don't?

The tree-length posterior estimates are affected by the inability to include the Jacobian adjustment between the embedding space and tree space, so they will not converge to the same posterior. This is discussed at the end of the "Fidelity" discussion and in supporting information S3 "Impact of Normal Priors". With four chains running on each MCMC, we do not necessarily suspect that chains are stuck in the same local optimum, but that being in any local optimum (or optima for each chain) may lead to only a subset of trees being explored. This is consistent with a higher deviation in splits. It is also possible that the Jacobian adjustment is systematically more significant with lower curvature. However, this is not consistent with the data. The trend of reduced variance for lower curvatures did not happen for every dataset, Fig 3c. We have added a sentence in the "Fidelity" discussion to address this point.

> Unfortunately, I didn't have sufficient time to check the Jacobians that you mention, but those are good places to check if the MCMC algorithm works correctly (which I'm suspecting is not).

We have double-checked these calculations. From section S3, we know that the Jacobian is responsible for the differing results. Section S3 presents an alternative prior that alleviates this issue.

> My final and main remark targets the actual improvement using your approach. In the introduction, you state that the current MCMC algorithms are performing poor because of the simplistic NNI and SPR algorithms. So how much does your approach improve over these well established and existing methods? It would be nice to see improvements, but I couldn't find any. Does your approach convergence faster (in terms of CPU time)?

We have enhanced the section on "Navigating Tree Space" in the results to highlight the advantages of this approach. Using an embedding space gives this method the ability to make multiple SPR moves and branch length changes at once. Current methods can only perform these one at a time.

To your second question, Dodonaphy is currently implemented in Python with some speed up from Cython. MrBayes is a mature program written in c++ which, in general results in reduced CPU time. As a result, MrBayes is generally faster in terms of CPU time.
