# Supplementary material for: Fidelity of hyperbolic space for Bayesian phylogenetic inference
Source: PLoS Comput Biol. 2023 Apr 26;19(4):e1011084. doi: 10.1371/journal.pcbi.1011084 (PMC10166537; doi:10.1371/journal.pcbi.1011084)
Supplement: Attachment — Submitted filename: response_to_reviewers.pdf [file pcbi.1011084.s003.pdf]

We are glad to see the positive reception of our first set of corrections. We have addressed the reviewer's comments point-by-point below. We have largely addressed these points through improved notation and added extra explanation to some parts of the text.

Reviewer #1:

> I find this revised edition of the paper significantly easier to follow, and it much more clearly highlights the significant effort the authors have invested in this work. The reorganization makes it much faster to read, and I find the revised Algorithm 1 particularly helpful. I greatly appreciate the increased exploration of MCMC mixing and the exploration of the set of splits encountered.

Thank you for reviewing the paper.

> I have a few small comments which follow, but no serious objections to the manuscript as-is.

> I believe there is a typo in Algorithm 1, line 6. Should not "Pair-wise alignment distances" be "Pair-wise hyperbolic distances" as on line 10?

Typo corrected.

> In the section "Dodonaphy Algorithm":
>
> - While I think I get the broad strokes from Equation 4, I do not quite follow what exactly a tangent space of the origin is and what the new T is.

We have added a more explicit explanation of tangent spaces. We added more detail to explain what it means for a tangent space to be at the origin and how this works in the algorithm.

> - For readers like me who are not well-versed in hyperbolic geometry, I wonder if an extra sentence of explanation of Equation 3 could be added.

We have changed the paragraph surrounding Equation 3 to be more readable to a general scientist. For clarity, there is more emphasis on describing the spaces involved.

> - Additionally, the use of T as a tangent space, where it had been a tree, and the introduction of \mathcal{T} for the tree is jarring.

We have changed the notation for the tangent space from T \mathbb{H} to \mathbb{T} \mathbb{H} both here and in the appendix. This should prevent any confusion with using T for a tree. On lines 163-164, we also refresh the notation T for a tree in comparison to the notation for the space of trees \mathcal{T}^n.

> Figure 2:
>
> - I like the replacement for panel B and find it much more interpretable.
> - The transparent diamonds, which were visible in the previous version of the MS/figure, seem to have disappeared?

The figure has been correctly saved to show the transparent diamonds, as in the original version.

> - Color labels for A/D would be nice (also for the similar figure in the appendix)

We have added a corresponding description to the captions of these figures.

> - Something seems to have gone wrong with the bar coloring for the two red points in B (also in the appendix)

The red dots are meant to be there. They indicate that the confidence interval from Dodonaphy does not include the value from the golden runs of MrBayes. We have added an explanation of this in both figures' captions.

> Circa line 440: Not every reader may be familiar with cosh(), or cosh() of a matrix.

We meant to apply this function element-wise and have clarified this in the text. We have also extended equation 6 to include the effect of curvature.

> I'm probably missing something obvious, but I'm having some trouble following the dimensions int the paragraph between 439 and 440. D seems to be (d+2)x(d+2), while S is (d+1)x(d+1).

We have clarified in lines 448-449 how D is (1)x(d+1) vector and have now written it with a lowercase d^* so that only the matrices in Eq.6 have upper-case.

> Also, does "S_{ij} = z_i" mean "the rows of S are z"?

Yes, we've clarified this in the text and switched to using the notation S_{i*} = z_i.

> In the text of the appendix, most references to Figure/Fig X are lower case, which might be a typo.

These have been changed to upper case.
